# Pharmacokinetic and Bioequivalence Study of Eldecalcitol Soft Capsules in Healthy Chinese Subjects

**DOI:** 10.1002/cpdd.1159

**Published:** 2022-08-26

**Authors:** Ting Li, Feifei Sun, Yanping Liu, Lingping Su, Ping Shi, Xin Li, Shuqin Liu, Chenjing Wang, Linjing Shen, Yu Cao, Shumin Wang

**Affiliations:** ^1^ Phase I Clinical Trials Center The Affiliated Hospital of Qingdao University Qingdao China; ^2^ Wenzhou Haihe Pharmaceutical Co., Ltd Wenzhou China; ^3^ Pharmacy Department of Beijing Chao‐yang Hospital Capital Medical University Beijing China

**Keywords:** Eldecalcitol, bioequivalence, pharmacokinetic, safety, healthy subjects

## Abstract

Eldecalcitol is a novel active vitamin D_3_ 1,25(OH)_2_D_3_ derivative used in the treatment of osteoporosis. To investigate and compare the pharmacokinetic and bioequivalent profiles of two eldecalcitol soft capsule formulations at a single dose of 0.75 µg in healthy Chinese volunteers, we conducted a randomized single‐dose, open‐label, two‐period crossover study under fasting and fed conditions. Eligible subjects were randomly assigned to receive reference eldecalcitol soft capsules or test capsules in the first treatment period and to receive another formulation in the second period. Serial blood samples were collected for pharmacokinetic analysis. Adverse events were recorded. In total, 28 healthy subjects were enrolled in the fasting trial and 30 subjects were enrolled in the fed trial. The geometric mean ratios of the test formulation to the reference formulation for C_max_, AUC_0‐t_, and AUC_0‐∞_ were 94.2%, 94.0%, and 103.3%, respectively, under fasting conditions and 100.1%, 97.3%, and 96.0%, respectively, under fed conditions. No severe adverse events were observed. The results showed that the test and reference eldecalcitol formulations were bioequivalent and well tolerated in healthy Chinese subjects under fasting and fed conditions.

Osteoporosis is a bone disorder that increases a person's risk of fracture due to low bone mineral density, impaired bone microarchitecture/mineralization, and/or decreased bone strength. This asymptomatic condition often remains undiagnosed until it manifests as a low‐trauma fracture of the hip, spine, proximal humerus, pelvis, and/or wrist, which frequently leads to hospitalization.^1^, ^2^, ^3^ A cross‐sectional study conducted in a Chinese adult population in mainland China from December 2017 to August 2018 showed that in China the prevalence of osteoporosis among those aged 40 years or older was 5.0% among men and 20.6% among women. The prevalence of osteoporosis increased in association with increasing age and was significantly higher among women compared with men.^4^ Another study conducted between July 2008 and June 2018 involving 75,546 participants who underwent routine physical examination in seven clinic centers from five provinces in China showed that currently a total of 10.9 million men and 49.3 million women in China are estimated to have osteoporosis. The age‐standardized prevalence of osteoporosis in China is estimated to be 6.5% and 29.1% for men and women aged 50 years and older, respectively.^5^

Eldecalcitol is an active vitamin D_3_ 1,25(OH)_2_D_3_ derivative for the treatment of osteoporosis that was first approved in Japan in 2011. With a hydroxypropoxy substitution at the 2β position of 1,25(OH)_2_D_3_, eldecalcitol shows strong effects on bone by improving bone metabolism, increasing bone mineral density, and preventing new vertebral fracture.^6^ Eldecalcitol is absorbed rapidly and eliminated gradually from serum with a mean time to maximum drug concentration (T_max_) of 3–4 hours and a mean half‐life (t_1/2_) of approximately 50 hours.^7^, ^8^ Pharmacokinetic exposure is dose‐proportional over the dose range of 0.1–1.0 µg, and t_1/2_ is not affected by the dosage. Eldecalcitol is metabolized to 1α,2β,25‐trihydroxyvitamin D3 in human small intestine and liver microsomes.^9^ Cytochrome P450 isoform 3A4 (CYP3A4) and sterol C4‐methyl oxidase‐like gene product (SC4MOL) contribute mostly to the metabolism of eldecalcitol.^10^, ^11^

Even though eldecalcitol has been well studied in Japan, there have been few studies in Chinese subjects, especially under fed conditions, to date. Moreover, although eldecalcitol soft capsules from Japan have been marketed in China, many Chinese patients cannot afford them, thus a generic equivalent is needed to meet the huge demand in the clinic. Here, to compare the pharmacokinetic and bioequivalent profiles of two eldecalcitol soft capsule formulations at a single dose of 0.75 µg under both fasting and fed conditions, we conducted this study in healthy Chinese volunteers.

## Methods

### Study Participants

A total of 58 healthy male and female Chinese subjects aged ≥18 years were enrolled in this randomized, open‐label, two‐period, crossover study, among which 28 participated under fasting conditions and 30 under fed conditions. All subjects weighed more than 50 kg with a body mass index between 19 and 28 kg/m^2^. All subjects did not meet the following exclusion criteria: (1) allergic to two or more substances or to experimental drugs; (2) a significant history of gastrointestinal inflammation/ulcer or other medical history affecting drug absorption or other diseases not appropriate to attend the trial; (3) presence of serious disease, major surgery or a history of trauma 3 months before screening; (4) use of any medication, including herbal medicine or healthcare products containing calcium, magnesium, or vitamin D, within 14 days before the first dose; (5) use of any investigational drug or product within 3 months prior to the first dose; (6) smoking more than five cigarettes a day in the last 3 months or cannot quit smoking during the study period; (7) alcoholic or drug abuser; (8) any abnormality with clinical significance of vital signs, physical examination, laboratory examination, and electrocardiograph (ECG) examination; and (9) consumption of any caffeine‐containing food or beverage, any beverage or food with abundant xanthine or any grapefruit or grapefruit‐containing juices within 48 hours prior to receiving study drug. All subjects were instructed to avoid pregnancy during the study period and for 6 months after the last dose.

### Study Design

This trial was conducted according to the Declaration of Helsinki, China Guidelines for Good Clinical Practice and local laws. The study protocol and informed consent form were approved by the Medical Ethics Committee of the Affiliated Hospital of Qingdao University. The objective and contents of the trial were fully explained to the participants. All subjects provided written informed consent before screening for eligibility. This trial was registered at the clinical trial register platform of the National Medical Products Administration (NMPA) (http://www.chinadrugtrials.org.cn) (No. CTR20201879).

A sample size of 28 for the fasting condition and 30 for the fed condition was estimated based on our preliminary results and the hypothesis of one‐side α = 0.05, β = 0.2, a bioequivalence limit of 80%–125%, and a drop‐out rate of 15%.

All eligible subjects were hospitalized on day −3 in the phase I clinical trials center of the Affiliated Hospital of Qingdao University to balance their diet before dosing. All subjects fasted overnight but without water restraints for at least 10 hours before the dosing day. All 28 subjects participating in the fasting trial were randomly assigned to two groups to receive a single dose of a 0.75‐µg reference eldecalcitol soft capsule (R; Edirol^®^, Chugai Pharmaceutical Co., Ltd, Fujieda City, Japan) or test capsule (T; Wenzhou Haihe Pharmaceutical Co., Ltd, Wenzhou, China) with approximately 240 mL of warm water in the first treatment period and to receive another formulation in the second treatment period. A 21‐day washout period occurred between the two treatment periods. For those who participated in the fed trial, the parameters were similar, except for having a unified breakfast with high fat and high calories 30 minutes before dosing. The total energy of the breakfast was 911.9 kcal, and 56.7% of the total calories were derived from fat. The meal consisted of 230 mL of milk, 100 g of apple pie, 110 g of sausage, 60 g of egg, and 10 g of butter with 150.0, 244.8, and 517.1 kcal derived from protein, carbohydrates, and fat, respectively.

All subjects were not allowed to drink water within the period between 1 hour before and after the dose was administered, and all subjects had a unified standard diet for their lunch and supper 4 and 10 hours after dosing. No other food or beverage intake was permitted except the provided diets. Cigarettes and alcohol were prohibited during the study.

### Blood Sampling and Pretreatment

Blood samples (8 mL) for the determination of eldecalcitol were collected predose and at 0.5, 1, 2, 2.5, 3, 3.5, 4, 4.5, 5, 6, 8, 10, 12, 15, 24, 36, 48, 72, 96, 120, 144, and 168 hours postdose in the fasting trial. In addition, for those participated in the fed trial, blood samples (8 mL) were collected predose and at 1, 2, 3, 4, 5, 6, 8, 10, 12, 14, 16, 24, 28, 36, 48, 72, 96, 120, 144, 168, and 192 hours postdose. Blood samples were kept at room temperature for at least 30 minutes and then centrifuged (4°C, 1700 *g*, 10 minutes) within 2 hours after sample collection. The separated sera were extracted into two labeled cryogenic vials for detection (approximately 2 mL) and back‐up (the remaining serum) and stored at −80°C.

### Sample Assay Method

Serum samples were first pretreated by the liquid–liquid extraction method and then analyzed for eldecalcitol by a validated liquid chromatography‐tandem mass spectrometry (LC–MS/MS) method.^12^ The chromatographic system consisted of an Acquity Ultra Performance Liquid Chromatography (UPLC) unit and a C_18_ column (150 × 2 mm). Mobile phases A and B were prepared based on the instructions of the assay and detection laboratory of Chongqing Denali Med Pharma. The UPLC was interfaced with an AB SCIEX Triple Quad 6500+ mass spectrometer. The electrospray ionization technique was employed using positive ion mode and multiple reaction monitoring mode. Eldecalcitol‐d6 was used as the internal standard.

The concentrations of the calibration standards were 10.0, 20.0, 50.0, 100.0, 300.0, 600.0, 900.0, and 1000.0 pg/mL. The lower limit of quantification (LLOQ) and upper limit of quantification (ULOQ) of this assay were 10.0 and 1000.0 pg/mL, respectively. Five levels of quality control (QC) samples were used to evaluate the within‐run and between‐run precision and accuracy. The LLOQ QC, low‐level QC, low‐mid‐level QC, mid‐level QC, and high‐level QC values were 10.0, 30.0, 80.0, 400, and 800 pg/mL, respectively. The maximum within‐run precision (expressed as coefficient of variation, CV%) of all the QC samples, with the exception of the LLOQ QC, was 11.4% and the accuracy (% deviation from theoretical level) was within −8.0 to 11.3. The maximum within‐run precision (CV%) of the LLOQ QC sample was 11.5% and the accuracy was within −3.0% to 12.0%. The maximum between‐run precision (CV%) of all the QC samples with the exception of the LLOQ QC was 9.4% and the accuracy was within −3.3 to 0.1. The maximum between‐run precision (CV%) of the LLOQ QC sample was 10.7% and the accuracy was 4.0%.

### Pharmacokinetic Analysis

Pharmacokinetic (PK) parameters were evaluated or calculated based on the serum concentration data of eldecalcitol through noncompartment analysis using WinNonlin (version 8.1 or above) software. The primary PK parameters were C_max_, AUC_0‐t_, AUC_0‐∞_, T_max_, t_1/2_, λ_z_, and AUC__%Extrap_. The C_max_ and T_max_ were obtained directly from the concentration–time data. The terminal phase rate constant (λ_z_) for eldecalcitol and the corresponding serum terminal phase t_1/2_ were calculated. AUC_0‐t_ was defined as the area under the concentration–time curve. AUC_0‐∞_ was defined as the area under the concentration–time curve from zero up to infinity with extrapolation of the terminal phase, and AUC__%Extrap_ was the area under the concentration–time curve extrapolated from the last measurable concentration to infinity and reported as the percentage of the total AUC. For those with AUC__%Extrap_>20%, descriptive statistical analysis was not performed for AUC_0‐∞_, t_1/2_, λ_z_, and AUC__%Extrap_. Of note, all beneath limit of quantification (BLQ) values were entered as zero and included as such in the calculation of means. Analysis of variance (ANOVA) was performed on the logarithmically transformed values of C_max_, AUC_0‐t_, and AUC_0‐∞_, in which model sequence, formulation, and period were used as fixed effects and subject nested within the sequence was used as the random effect. If the 90% confidence intervals of the geometric mean ratio (GMR) of the test formulation to the reference formulation were within the range of 80%–125%, the test formulation and reference formulation would be considered to be bioequivalent.

### Safety Assessment

All subjects who were randomized and received at least one dose of eldecalcitol were included in the safety analysis set (SS). Safety analysis was performed using SAS software (version 9.4 or above) based on SS data, including the incidence of adverse events (AEs) and corresponding descriptive statistics of clinical laboratory test values, vital signs, and electrocardiograms.

## Results

A total of 114 Chinese subjects were screened for this study. In total, 28 healthy subjects (24 males and four females) were enrolled in the fasting trial and 30 subjects (23 males and seven females) were enrolled in the fed trial. The demographic details are showed in Table S1.

All 28 subjects in the fasting trial completed the study, but one subject used multivitamin tablets after T_max_ in the second treatment period, which was considered an additive effect drug as mentioned in the protocol. No other protocol deviation was considered to significantly affect the results, and no subjects withdrew from this study under fasting conditions.

In the fed trial, all but two subjects completed the study. One subject withdrew from the study voluntarily when she completed the first treatment period trial, and the other withdrew early in the second treatment period due to a reported interfering drug combination with a rabies vaccine when the patient was accidently bit by a dog.

### Pharmacokinetics

All subjects in the fasting trial were included in the pharmacokinetic evaluation, except one subject who took multivitamin tablets. In the descriptive statistics, the subject's AUC_0‐t_ and AUC_0‐∞_ were not taken into account. According to the protocol, descriptive statistics of AUC_0‐∞_, t_1/2_, λ_z_, and AUC__%Extrap_ were not obtained from seven subjects from the test formulation group and four subjects of the reference formulation group for their AUC__%Extrap_ >20%.

**Figure 1:**
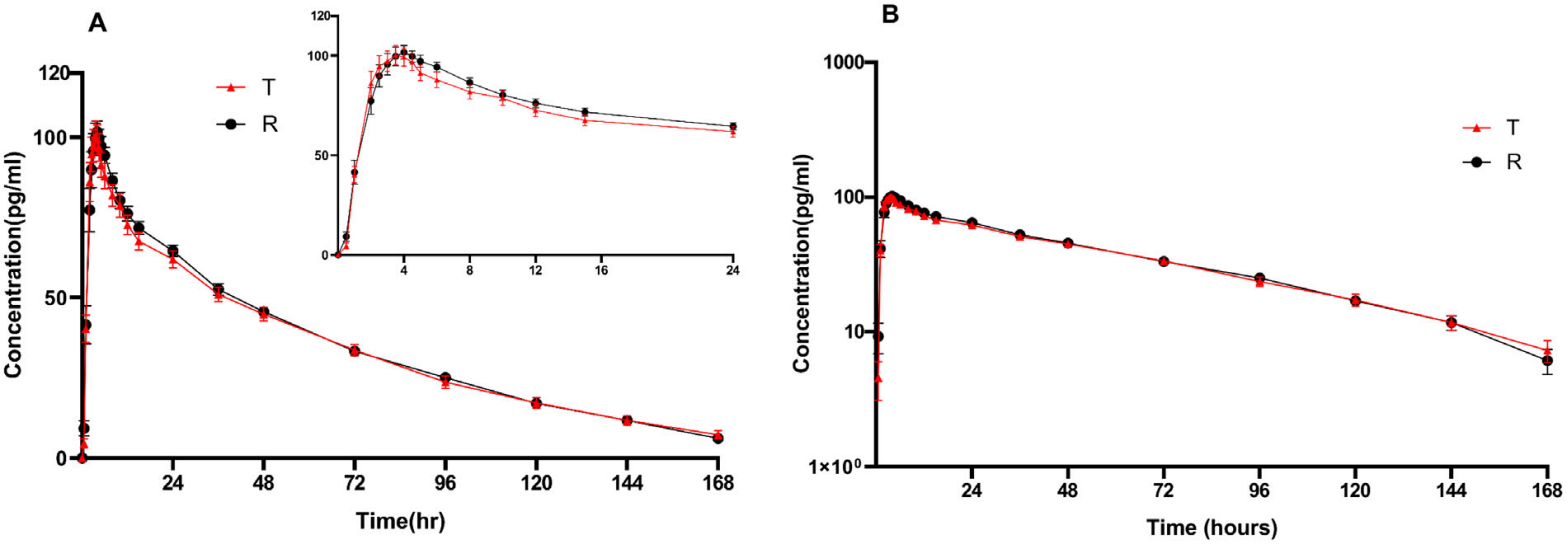
Serum concentration–time profiles (mean ± SE) of eldecalcitol after a single oral administration of a 0.75‐µg eldecalcitol soft capsule in healthy Chinese subjects under fasting conditions. (A) Linear scale; (B) semi‐logarithmic scale.

The mean serum concentration–time profiles of eldecalcitol following a single dose of 0.75 µg eldecalcitol are shown in Figure 1 and the PK parameters are shown in Table 1. Compared to the reference formulation, the relative bioavailabilities of eldecalcitol in C_max_, AUC_0‐t_, and AUC_0‐∞_ were all in the range of 80%–125% (shown in Table 2). ANOVA results revealed statistically significant differences in C_max_, AUC_0‐t_, and AUC_0‐∞_ were noted in subjects nested within the sequence (*P* < .05), but no significant difference in sequence, period and formulation was observed (*P* > 0.05).

**Table 1:** Comparison of PK Parameters of Eldecalcitol in Healthy Chinese Subjects After a Single Oral Administration of a 0.75‐µg Eldecalcitol Capsule (Test Formulation or Reference Formulation) Under Fasting and Fed Conditions

	Test Formulation (T)		Reference Formulation (R)	
PK Parameters (unit)	N	Mean ± SD (CV%)	N	Mean ± SD (CV%)
Fasting				
C_max_ (pg/mL)	28	103.6 ± 24.4 (23.6)	28	107.3 ± 15.7 (14.7)
AUC_0‐t_ (h*pg/mL)	27	5559.5 ± 1768.0 (31.8)	28	5716.9 ± 1276.2 (22.3)
AUC_0‐∞_ (h*pg/mL)	20	7205.3 ± 1324.3 (18.4)	24	6762.8 ±1465.5 (21.7)
T_max_ (h)^a^	28	3.5 (2.5,5.0)	28	3.5 (2.5, 6.0)
t_1/2_ (h)	21	53.3 ± 8.9 (16.7)	24	50.8 ± 8.9 (17.6)
Fed				
C_max_ (pg/mL)	29	99.5 ± 16.5 (16.6)	30	99.0 ± 14.0 (14.2)
AUC_0‐t_ (h*pg/mL)	28	6051.1 ± 1509.6 (24.9)	30	6134.1 ± 1278.2 (20.8)
AUC_0‐∞_ (h*pg/mL)	27	7042.3 ± 1631.6 (23.2)	28	7293.6 ± 1359.1 (18.6)
T_max_ (h)^a^	29	10 (2.00, 14.00)	30	10 (2.00, 36.00)
t_1/2_ (h)	27	50.5 ± 9.6 (19.1)	28	56.1 ± 10.6 (18.9)

CV, coefficient of variation.

a T_max_ (h) is expressed as median (minimum, maximum).

**Table 2:** Parameters in Bioequivalence Evaluation Under Fasting and Fed Conditions

	T		R		T/R		
Parameters (unit)	N	GeoLSM	N	GeoLSM	Ratio (%)	90%CI (%)	Intra‐Subject CV (%)
Fasting							
C_max_ (pg/mL)	28	99.9	28	106.1	94.2	88.4–100.3	13.9
AUC_0‐t_ (h*pg/mL)	27	5229.9	28	5561.9	94.0	86.5–102.2	18.1
AUC_0‐∞_ (h*pg/mL)	20	6782.7	24	6565.1	103.3	98.8–108.1	8.0
Fed							
C_max_ (pg/mL)	29	98.1	30	98.0	100.1	95.4–105.0	10.7
AUC_0‐t_ (h*pg/mL)	28	5838.2	30	6002.6	97.3	93.4–101.3	8.9
AUC_0‐∞_ (h*pg/mL)	27	6826.7	28	7107.8	96.0	92.5–99.7	7.9

GeoLSM, geometric least square mean.

**Figure 2:**
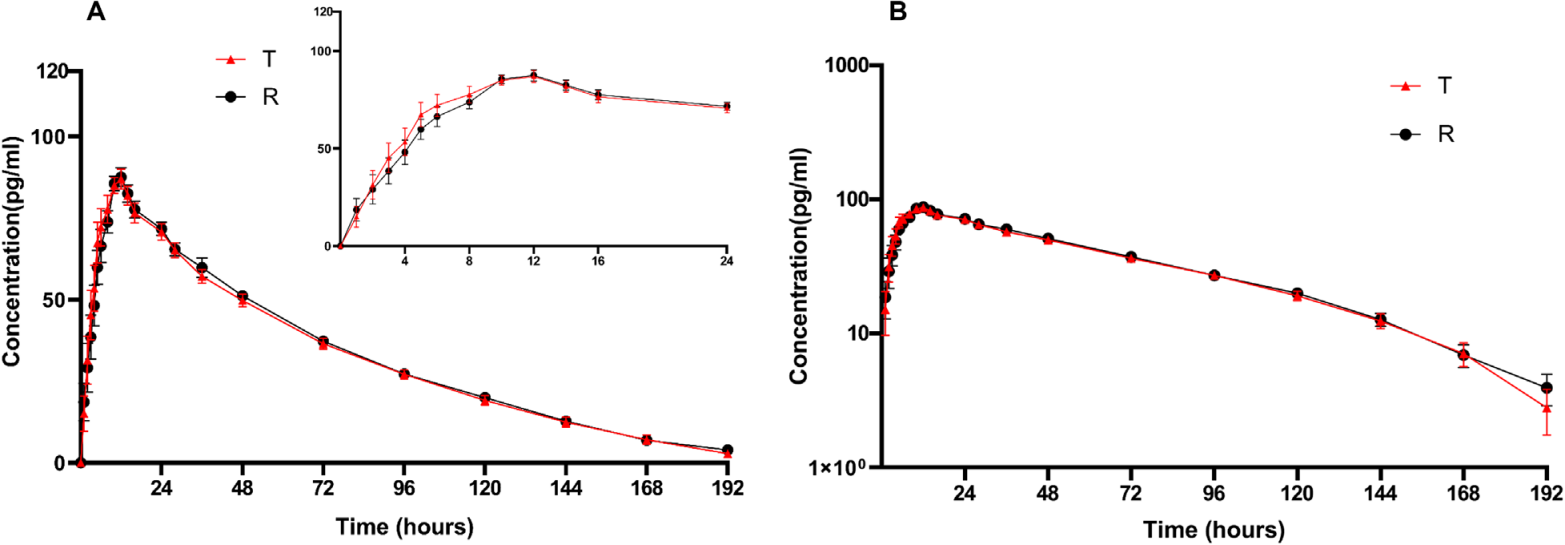
Mean serum concentration–time profiles (mean ± SE) of eldecalcitol after a single oral administration of a 0.75‐µg eldecalcitol soft capsule in healthy Chinese subjects under fed conditions (A) Linear scale; (B) semi‐logarithmic scale.

In the fed trial, all subjects were included in pharmacokinetic and bioequivalence evaluation, except the one patient who withdrew. Descriptive statistics of AUC_0‐∞_, t_1/2_, λ_z_, and AUC__%Extrap_ were not performed in two subjects from the test formulation group and two subjects from the reference formulation group based on AUC_‐%Extrap_ >20%. The mean serum concentration–time profiles of eldecalcitol following a single dose of 0.75 µg of eldecalcitol under fed conditions are shown in Figure 2. The PK parameters are shown in Table 1 and the bioequivalence analysis is shown in Table 2. ANOVA results revealed statistically significant differences in C_max_, AUC_0‐t_, and AUC_0‐∞_ in subjects nested within the sequence (*P* < 0.05), but no significant difference in sequence, period, and formulation was observed (*P* > 0.05).

### Safety

No subjects discontinued this study due to an AE, and no serious AE was reported. In the fasting trial, seven subjects experienced a total of seven AEs and five subjects experienced five adverse reactions (ADRs). The ADRs included increased triglyceride (3.6%), diarrhea (3.6%), oral ulcer (3.6%), eczema (3.6%), and increased aspartate aminotransferase (3.6%), all of which were mild. In the fed trial, seven subjects experienced a total of 11 AEs and five subjects experienced six ADRs. The ADRs included increased leucocyte count (3.3%), increased hemobilirubin (3.3%), increased triglycerides (3.3%), ventricular extrasystole (3.3%), and hypohemia (3.3%). The ADRs of increased hemobilirubin and increased triglycerides were classified as medium and all the others were mild.

## Discussion

This randomized, open‐label, two‐period, crossover study was conducted to compare the pharmacokinetic and bioequivalent profiles of two eldecalcitol soft capsule formulations at a single dose of 0.75 µg under both fasting and fed conditions in healthy Chinese volunteers. For most dosage forms of the release drug intended to be systemically available, a two‐period, two‐sequence, two‐treatment, single‐dose, crossover study using healthy subjects is recommended.^13^ To completely reflect the whole process of drug disposal in vivo, the PK sampling window should cover three to five half‐lives, sampling should continue until the tested drug concentration reaches 1/10–1/20 of C_max_, or AUC__%Extrap_ should generally be less than 20%. After administration, the therapeutic blood concentration of eldecalcitol is very low, with a long half‐life of approximately 50 hours. In this study, we used the LC–MS/MS method to detect the eldecalcitol concentration in the samples. LC–MS/MS is a widely used sensitive quantitative detection technique. In our study, although the sample collected at 168 hours after administration did not cover three to five half‐lives, only 20% (11/55) of participants in the fasting study and 7% (4/59) in the fed study of AUC__%Extrap_ exhibited values greater than 20%, which generally describes the pharmacokinetic behavior of eldecalcitol. Regarding the C_max_, AUC_0‐t_, and AUC_0‐∞_ of eldecalcitol, the analysis of variance revealed no significant differences between the two eldecalcitol preparations, and the 90% confidence interval for the GMRs fell within the predefined acceptance range of 80%–125%. The results fit the claim of bioequivalence, and the equivalent formulations are typically considered to be therapeutically equivalent.^14^

In a previous study conducted in healthy Japanese male subjects, a single oral dose of 0.75 µg of eldecalcitol was administered to 32 healthy male volunteers while fasting. After a washout period ranging from 14 to 21 days, 15 of these subjects also took another single oral dose after breakfast to investigate the effect of food. The results showed that under fasting conditions, the C_max_ of eldecalcitol was reached in 3.4 hours at approximately 99.8 pg/mL (with a half‐life of 53.0 hours). Under fed conditions, the C_max_ was reached in 5.7 hours at approximately 95.4 pg/mL.^7^ Another phase I study conducted in 24 healthy male Chinese subjects showed that after a single dose of 0.75 µg of eldecalcitol taken orally under fasting conditions, the C_max_ of eldecalcitol was reached in 3 hours (median) at 94.4 pg/mL and the half‐life was 53.1 hours.^8^

In this study, after a single dose of 0.75 µg of eldecalcitol administered under fasting conditions, the C_max_ of eldecalcitol was reached in 3.5 hours (median) at approximately 103.6 pg/mL (with a half‐life of 53.3 hours) in the test formulation and 107.6 pg/mL (with a half‐life of 50.8 hours) in the reference formulation. Under fed conditions, the C_max_ of the test formulation was 99.5 pg/mL and for the reference formulation the value was 99.0 pg/mL. The absorption rate results are similar to those previously reported in healthy Chinese and Japanese male subjects.^7^, ^8^ However, the LLOQ used in the previous studies was 25 pg/mL. In this study, the LLOQ was 10 pg/mL, which was considerably more accurate and precise for detection. There was no comparability in the extent of absorption.

Although the food effect on eldecalcitol systemic exposure was not the objective of our study, we made a comparison based on the parallel comparison of pharmacokinetic parameters between the fasting and fed arms of the study. The time courses of serum eldecalcitol concentrations with administration while fasting and after a meal were similar. Food has no effect on eldecalcitol, which is consistent with the results reported in Japanese subjects.

An interim report of a postmarketing observational study showed that the most common ADRs reported included hypercalcemia/increased blood calcium, renal impairment, abdominal discomfort, constipation, pruritus, thirst, nausea, eczema, rash, and vertebral compression fracture.^15^ In a phase I study conducted in healthy Chinese male subjects, the AEs observed included herpes zoster infection, upper respiratory tract infection, dry pharynx, rash, and diarrhea. Furthermore, some laboratory abnormalities were reported, including an increase in γ‐glutamyltransferase, alanine aminotransferase, aspartate aminotransferase, leucocyte count, neutrophil count, triglyceride, and lactate dehydrogenase, a decrease in hemoglobin and glucose, urine leukocyte positivity, abnormal urine sediment, and urobilinogenuria.^8^ Most of the ADRs that occurred in our study were formerly reported, except for oral ulcers and ventricular extrasystole. In general, both the test and reference eldecalcitol soft capsules showed good tolerance.

## Conclusion

This study reported the pharmacokinetics and safety of eldecalcitol in Chinese subjects under both fasting and fed conditions using a sensitive detection method with a much low LLOQ. The results demonstrate that the test formulation and reference formulation were bioequivalent and that both formulations were well tolerated.

## Funding

This work was supported by grants from the National Major Scientific and Technological Special Project for Significant New Drugs Development (2020ZX09201‐018, 2017ZX09304‐024) and the Natural Science Foundation of Shandong Province (ZR2019MH101).

## Conflicts of Interest

The authors report no conflicts of interest.

## Supporting information

Supporting information
