# Supplementary material for: Pharmacokinetic and Bioequivalence Study of Eldecalcitol Soft Capsules in Healthy Chinese Subjects
Source: Clin Pharmacol Drug Dev. 2022 Aug 26;11(12):1474–80. doi: 10.1002/cpdd.1159 (PMC10087228; doi:10.1002/cpdd.1159)
Supplement: Supplementary file 1 — Supporting information [file CPDD-11-1474-s001.docx]

**Supplemental Table 1**. Baseline demographic characteristics (mean ±standard deviation)

| Sequence |  | Fasting group | | |  |  | Fed group | | |
| --- | --- | --- | --- | --- | --- | --- | --- | --- | --- |
|  | T-R(n=14) | R-T(n=14) | Total(n=28) | P-value |  | T-R(n=15) | R-T(n=15) | Total(n=30) | P-value |
| Age(years) | 29.9±5.8 | 31.0±5.8 | 30.4±5.7 | 0.49 |  | 31.4±9.6 | 30.5±6.8 | 30.9±8.2 | 0.83 |
| Male/Female | 13/1 | 11/3 | 24/4 | 0.56 |  | 12/3 | 11/4 | 13/7 | 0.91 |
| Height(cm) | 171.5±6.1 | 168.2±7.6 | 169.8±6.9 | 0.41 |  | 169.5±8.8 | 166.0±7.4 | 167.8±8.2 | 0.16 |
| Weight(kg) | 68.0±8.1 | 67.4±11.0 | 67.7±9.5 | 0.95 |  | 70.0±7.4 | 63.0±8.4 | 66.5±8.5 | 0.03 |
| BMI(kg/m^2^) | 23.1±2.1 | 23.8±2.8 | 23.4±2.4 | 0.46 |  | 24.4±2.3 | 22.8±2.3 | 23.6±2.4 | 0.10 |
